# Navigating post-ICU care: understanding family members’ experiences - a qualitative study

**DOI:** 10.1080/21642850.2024.2415394

**Published:** 2024-10-11

**Authors:** Matteo Danielis, Alessandro Garau, Dina Molaro, Sara Gentilini, Marika Rosset, Serena Giorgino, Federica Vuerich, Renzo Zanotti, Lorenza Entilli

**Affiliations:** a Laboratory of Studies & Evidence Based Nursing, Department of Cardiac, Thoracic, Vascular Sciences and Public Health, University of Padova, Padova, Italy; b Department of Emergency, Academic Hospital of Udine, Udine, Italy; c Department of General Psychology, University of Padua, Padova, Italy

**Keywords:** Family members, caregivers, intensive care unit, nursing, quality of life

## Abstract

**Background:**

Comprehending and addressing the needs of caregivers during the post-intensive care unit (ICU) phase is vital for establishing sustainable support systems and improving the overall quality of life (QoL) for both patients and caregivers.

**Objective:**

To explore the experiences of family members (FMs) caring for loved ones three-months after ICU discharge and their related QoL.

**Methods and measures:**

A qualitative, descriptive research was conducted. Participants were recruited from two general ICUs in an Italian Academic Hospital. Data collection lasted two months and was performed with telephonic interviews led by ICU nurses. Thematic analysis was conducted using a hybrid approach, incorporating both deductive and inductive coding strategies. This process has been facilitated by Atlas.ti software.

**Results:**

Twenty-four FMs participated, representing a diverse range of familial relationships with the patients. Thematic analysis revealed four overarching themes: 1) QoL underwent transformations; 2) Positive emotions laden with significance; 3) Supporting role taken on by a caregiver; and 4) Life’s transience through the meaning-making of the illness event. These themes highlighted the multifaceted nature of the caregiving experience.

**Conclusions:**

This study provides valuable insights into the challenges and dynamics faced by FMs following ICU discharge. Findings underscore the importance of addressing environmental challenges, cultivating positive emotions, and strengthening caregiver-patient relationships to enhance the caregiving experience and promote overall QoL. FMs can adapt their personal concepts and reach their full potential by learning to coexist with the demanding role of caregiver and achieve a new level of resilience and fulfillment.

## Introduction

Family members (FMs) assume a pivotal role within the Intensive Care Unit (ICU), contributing not only emotional support and practical assistance to the patient, but also engaging in collaborative efforts with healthcare professionals to optimize the delivery of care. FMs, who are spouses, relatives, or friends of sick, disabled, and dependent individuals, provide unpaid care (Cruz et al., 2023). This support, lasting months or even years, includes various physical, social, emotional, and financial aspects. Approximately 95% of patients within the ICU necessitate the involvement of a surrogate decision-maker, commonly identified as a FM decision maker, to actively participate in communication and decision-making processes (Davidson et al., 2017; Petrinec & Daly, 2016). Current literature advocates for the involvement of family members from the onset of their ICU experience to effectively prepare them for post-discharge challenges (Avgeri et al., 2023; Danielis et al., 2023; McAndrew et al., 2020). Then, upon discharge from the ICU, the commitment of FMs endures, as they play a key role in the patient's recovery. As the post-ICU period for patients and families is characterized by a complex interplay of physical, emotional, and logistical factors, the quality of life (QoL) for these FMs is profoundly impacted by the intensive caregiving responsibilities and the associated stressors. The cumulative impact of familial exposure to critical illness may give rise to a phenomenon identified as Post-Intensive Care Syndrome-Family (PICS-F) (Davidson et al., 2012). Understanding and addressing the needs of caregivers in this critical post-ICU phase are essential for enhancing their overall QoL and ensuring sustainable support systems are in place (Zante et al., 2020).

The first three months after ICU discharge are usually critical, as patients – and consequently their FMs – undergo profound transformations and experience new symptoms, leading to a period of adaptation to their new lives. During this time, the post-ICU experience is often marked by anxiety and stress. Patients frequently report difficulties sleeping, a strong need for normal sleep, and being tormented by nightmares after discharge (Altman et al., 2017; Chahraoui et al., 2015). Prolonged bed rest during critical illness can cause lasting muscle discomfort and pain, even three months post-ICU. Another study found that functional dependence initially increased but decreased within one to three months, impacting QoL in terms of mobility, personal care, habitual activities, pain, and anxiety/depression (Lobato et al., 2023).

The FMs QoL is contextualized within the specific domain of helping a loved one with personal care, household duties, and various activities of daily living. The conceptual framework proposed by Martin and colleagues to define caregiver QoL delineates four fundamental dimensions, namely: physical capacity (e.g. energy and fatigue), psychological state (e.g. beliefs and values), social relations (e.g. social support), and environment (e.g. financial resources) (Martin et al., 2021). The impact of family care has been investigated in qualitative research in recent years. As described in a qualitative study exploring the experiences of 14 relatives of critically ill patients discharged from two non-COVID ICUs during the COVID-19 pandemic, FMs experienced mixed emotions initially. At home, limited community services often led them to seek private healthcare support, exacerbating care issues and isolating the patient-relative dyad due to restricted hospital access and limited formal and informal care options (Danielis et al., 2022). This exploration has suggested that the potential decline in patients’ QoL may significantly affect their relatives as well. Therefore, further exploration is warranted to investigate the post-ICU discharge implications on the care requirements of the dyad (patient + FM). In this line of research, the study of Vester and colleagues has also delved into the experience of everyday life and recovery after critical illness of seven FMs and 12 patients (Vester et al., 2022). Critical illness and the resulting physical, psychological, and cognitive care challenges create new difficulties for patients and their relatives in everyday life. This experience impacts their self-perception, family dynamics, and ability to resume activities such as work and social engagements (Vester et al., 2022). From these findings, it emerged the significance of augmenting the established domains in PICS-F with a social health dimension to better determine QoL of FMs. This is supported by evidence indicating that FMs of ICU patients experience a significant decline in QoL during the first year following the patient's admission to the ICU (Alfheim et al., 2019).

Despite the imperative to address the diverse needs of FMs following patients’ discharge from the ICU, existing studies vary in follow-up timing and historical context (e.g. COVID vs. non-COVID periods), and their qualitative analyses are influenced by the specific contexts in which they are conducted. This study aims to provide a consistent and contextualized understanding of caregivers’ experiences during the post-discharge period, specifically within the Italian context, after the COVID pandemic has ended. For patients, the insights gained can lead to improved post-ICU care, enhancing recovery and overall wellbeing. For families, understanding the post-ICU challenges enables better support for their loved ones and reduces caregiver stress. For healthcare organizations, the study provides valuable data to optimize resources, develop targeted interventions, and improve patient and FMs outcomes, ultimately leading to more efficient and effective healthcare delivery.

## Method

### Study design and aim

This qualitative and descriptive research, employing interviews, facilitated the elucidation of participants’ experiences, providing an authentic account of events (Sandelowski, 2010). The consolidated criteria for reporting qualitative research (COREQ checklist) were considered in the preparation of the manuscript (Tong et al., 2007).

The aim of this study was to investigate the experiences of FMs in the caregiving role following the discharge of their loved ones from an ICU, with a particular focus on how these experiences impact their QoL. Our main research question was as follows: ‘What are the day-to-day experiences of FMs caring for a loved one at home after ICU discharge, and how do these experiences intersect with the perceived QoL of the caregiver?’.

### Participants

Participants, both patients and their relatives, were enlisted from two general ICUs located within an Academic Hospital in the North-Eastern region of Italy, which boasts over 1,000 beds. Purposive sampling (Benoot et al., 2016) was employed to select a single relative of each adult patient (≥ 18 years) with a length of stay (LOS) of three days or more in the ICU between December 1, 2022, and February 28, 2023. Inclusion criteria comprised: (a) being a spouse, blood relative (related by direct genetic descent or shared ancestry such as parents, siblings, children, grandparents, aunts, uncles, nieces, nephews, and cousins), or the identified next of kin; (b) age 18 or older; and (c) willingness to visit the patient daily. Exclusions encompassed (a) relatives of patients diagnosed by the medical doctor as terminally ill ( = a progressive or untreatable disease condition from which death is expected in the near future) and (b) those experiencing an unanticipated discontinuation of daily visits to their loved ones in the ICU. Data saturation was achieved (Vasileiou et al., 2018), as independently determined by two researchers (MD, AG). The participants received no financial compensation, with clear elucidation that their involvement in the study was independent of any potential access to care.

### Data collection

Three months after discharge from the ICU, telephone interviews were conducted to find out about the experiences of relatives. These interviews took place between March and May of 2023, concluding on May 30th. All telephonic communications and subsequent audio-recorded interviews were administered by a team of researchers (see authors), comprised of registered nurses (both male and female) working in the ICUs at the time of the study. The mean interview duration averaged 18 minutes, ranging from 10 to 30 minutes. Each interview initiated with an open-ended question: ‘What emerged from the time of discharge to the present?’. Subsequent to this, principal inquiries encompassed: ‘In what manner has your quality of life evolved subsequent to hospitalization in the ICU? Currently, how do you undertake the care of your loved one?’. Furthermore, probing queries (e.g. ‘What is the intended meaning?’ and ‘Could you elaborate on this concept?’) were employed to elucidate experiences or redirect focus to the core subject. No pre-constructed tool was used; instead, the interviews were conducted using the same methodology as a previous study (Danielis et al., 2022). Participants were explicitly encouraged to articulate their experiences comprehensively. It is pertinent to note that the interview questions were not disclosed to the relatives in advance.

### Analysis

Initially, all audio-recorded interviews underwent verbatim transcription utilizing Amberscript® software (retrieved on August 16, 2023, from https://www.amberscript.com). Subsequently, a researcher (MD) meticulously reviewed the transcripts to identify and rectify any transcription errors. Following this, all researchers independently and thoroughly read the texts to gain a comprehensive understanding of the experiences. Finally, the data were imported into the qualitative data analysis program ATLAS.ti® version 22 for further analysis.

The analysis adhered to Braun and Clarke's (2013) thematic analysis (TA) methodology (Braun & Clarke, 2013), with a hybrid approach to TA, where initial codes were driven by both data per se and theories (Xu & Zammit, 2020). Initially, a deductive coding approach was employed, drawing upon findings from a relevant article on QoL (Martin et al., 2021) and another with a comparable population and research question to the present study (Danielis et al., 2022). Moreover, Cutrona and Suhr’s social support category system, which involves five general categories of social support: (a) informational, (b) emotional, (c) esteem, (d) social network support, and (e) tangible support, was considered (Cutrona & Suhr, 1992). As social support is an important factor influencing QoL, the use of Cutrona and Suhr’s framework allowed for an in-depth analysis of how support mechanisms (or lack thereof) influence caregivers’ QoL. The choice of this framework was carefully aligned with the study’s aim to ensure a robust and nuanced examination of the caregiving experiences in post-ICU period. Subsequently, an inductive coding phase was introduced to uncover additional, emergent themes within the data. Taking a bottom-up approach, the authors reflected on their personal and professional experiences as nursing research professional and health psychologist researcher. This dual approach aimed to provide a comprehensive understanding of the narratives related to testing, incorporating both established categories and unanticipated nuances for a more detailed exploration of the data.

The authors adhered to all stages of thematic analysis. They initiated the process by becoming familiar with the data and then proceeded to the preliminary coding phase. One of the authors (MD) conducted the initial coding, which was subsequently reviewed and discussed iteratively with a second author (LE). Preliminary code definitions were documented in code memos that included usage criteria and transcript passages that exemplified the use of each code. These, were discussed within the two authors to ensure a common understanding of the code system. When consensus on the code system was reached, one author (AG) completed the data analysis by selecting specific codes for evaluation, performing in-depth analysis and interpretation and determining the analytical approach and presentation of results. The interpretation of the data was supported by argumentative substantiation based on the original data and interpersonal validation. The coding process reached its conclusion when both authors reached agreement on the final coding and themes.

### Ethics

As part of a research project focused on assessing family contribution on patients’ QoL ( = FENICE, Family ENgagement in Intensive Care Environments, approved by the Regional Ethics Committee of Friuli Venezia Giulia Region, Italy [number: CEUR-2020-Sper-012]), we have approached FMs over the phone, to gain valuable perspectives and insights. While this approach deviates from the original FENICE protocol, we took utmost care to ensure transparency, respect for participants’ autonomy, and adherence to ethical principles set by the Declaration of Helsinki. The protection of personal data was guaranteed by observing Regulation (EU) 2016/679 of the European Parliament. Moreover, participants were provided with comprehensive verbal information about the study objectives and procedures, and the voluntary nature of their participation. They provided oral consent to participate in the study, which was documented through audio recordings. In addition, all participants gave their approval to be audio-recorded. To safeguard participants’ anonymity, qualitative data from family members were recorded using consecutive numbers instead of personal identifiers (i.e. Family Member 1, FM1). In accordance with the protocols of the ethic committee for the collection of informed consent, the participants were explained that the data collected will be kept, duly anonymized, for five years and then deleted and that such data will not be used in any way for evaluations relating to their treatment path.

## Results

In our study, we initially approached a total of 40 family members who met the inclusion criteria. Out of these, we successfully conducted interviews with 24 participants. The FMs exhibit a nearly balanced demographic distribution, with 14 females (58.3%) and 10 males (41.7%), and an age range spanning from 26 to 76 years. In terms of relationships to the patient, there were ten daughters/sons (41.7%), five siblings (20.8%), including brothers and sisters, and three parents (12.5%), combining mothers and fathers. Additionally, there were six spouses (25.0%), evenly distributed between wives and husbands. Demographic information is given in Table 1.

**Table 1.** Participant demographics.

ID	Gender	Age (years)	Relationship to patient
FM 1	F	64	Mother
FM 2	F	46	Daughter
FM 3	M	73	Brother
FM 4	F	56	Wife
FM 5	F	48	Daughter
FM 6	M	56	Son
FM 7	F	52	Daughter
FM 8	F	60	Wife
FM 9	M	26	Son
FM 10	F	59	Daughter
FM 11	F	55	Daughter
FM 12	F	29	Daughter
FM 13	F	71	Sister
FM 14	M	61	Brother
FM 15	M	52	Son
FM 16	M	70	Father
FM 17	F	51	Daughter
FM 18	M	52	Brother
FM 19	M	55	Husband
FM 20	F	61	Mother
FM 21	M	76	Husband
FM 22	M	61	Husband
FM 23	F	47	Sister
FM 24	F	51	Wife

ID = identification; FM = family member; M = male; F = female.

**Figure 1.**
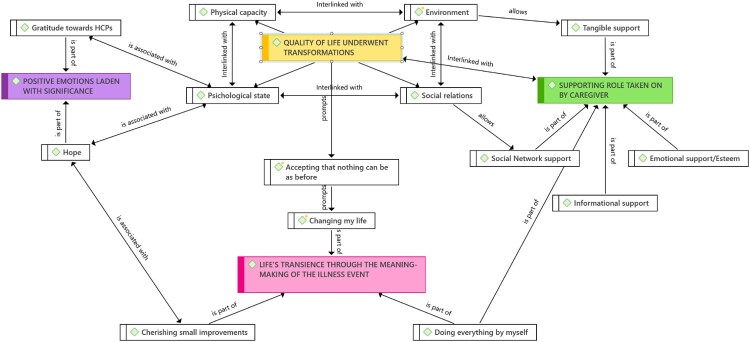
Concept map of codes and themes.

Our qualitative analysis revealed four distinct themes: 1) Quality of life underwent transformations; 2) Positive emotions laden with significance; 3) Supporting role taken on by a caregiver; and 4) Life’s transience through the meaning-making of the illness event. Table 2 illustrates the structure of the themes found, while Figure 1 depicts the relationships and interconnections between codes and themes.

**Table 2.** Themes with their constituent codes and representative quotations.

Theme	Code	Quotation
**1. Quality of life underwent transformations**	Environment	FM10: To intervene and help my mother, we have to drive about 40 minutes, so we can't be there immediately on the spot.
	Physical capacity	FM7: Honestly, right now, as I told my colleague, I cannot wait for her to come home because I’m a bit tired.
	Social relations	FM8: She still has her two grandchildren, she has her daughters, and then on Saturday, the grandchildren also came to sleep over to keep her company.
	Psychological state	FM8: I could not participate in the visit because I was notified only a few hours before … The unpleasant feeling that we family members had is that of feeling guilty, even though we shouldn't have.
**2. Positive emotions laden with significance**	Hope	FM9: Now I am, let's say, calm, I am positive, and I am moving forward, I have no problems.
	Gratitude towards HCPs	FM1: I can say thank you to everyone, both the hospital in [city] and the intensive care unit, who saved her
**3. Supporting role taken on by a caregiver**	Informational support	FM10: Clearly, on a bureaucratic level, yes, all the things he managed previously, we have to manage them together […] things like bills, managing the household.
	Emotional support/Esteem	FM1: Today I said to S. [the patient], ‘S., just help me with the vacuum cleaner for a moment, stand up straight’ and she does it.
	Social network support	FM6: Yes, it is going a little better. Yes, yes, she speaks. From the point of – There has been a decline, but let us say she recognizes us and participates at her own pace, let us say at her own pace, she participates in social activities.
	Tangible support	FM10: Together with our family, basically, my husband, my sister-in-law, yes, we coordinate, well, it also depends on work, and we allocate the various availabilities.
**4. Life’s transience through the meaning-making of the illness even**	Cherishing small improvements	FM7: she is still bedridden, rightly so, because honestly, they removed the tracheostomy tube yesterday, so, um, she is still mostly eating smoothies, so she does not have – in my opinion, she does not have all the strength she could have to walk or get up yet, I hope.
	Changing my life	FM4: The workload has increased for me. […] I try to stimulate him because I say, ‘If we both give up here, it's over, it is all over.’
	Doing everything by myself	FM1: – (*nurse*) However, do you manage to do everything alone at home? – Yes. – (*nurse*) You don't let anyone help you? – No. Absolutely
	Accepting that nothing can be as before	FM2: We, let's say, had to grit our teeth, keep going, because what else could I do? So, there, I cannot let my dad see me cry, I cannot let the little ones see me, so, uh, we have to fight. She is alive, she is here with us, and I hope she stays. And well, that's it. Life has been cruelled like that.

FM = family members; HCPs = healthcare professionals.

### Quality of life underwent transformations

From our qualitative interviews, a prominent theme emerged, encapsulated as ‘Quality of life underwent transformations.’ This theme signifies profound changes in individuals’ well-being, comprising four intricate codes.

The first code, environment, examines changes in surroundings, acknowledging the influence of external factors on daily life due to the new tasks and environmental changes required by the recovery of the family member. A caregiver described all the changes needed to host her father in her house, with the associated architectural difficulties.

FM7: I had to rearrange and prepare the bed for him downstairs.

The second, physical capacity, is often linked to these environmental changes and delves into the nuanced impact on the bodily functions of the caregivers, exploring shifts in health and mobility. Participants reported a draining physical and psychological tiredness, almost difficult to verbalize and dangerously close to fatigue.

FM1: I’m not tired, more. Much more than that … 

Social relations, the third code, unveils alterations in interpersonal connections, emphasizing the evolving dynamics of relationships. It emerges that the strain on the FMs’ social relationships, as they need to allocate their time and attention across different persons, emphasizing the challenges of maintaining social connections and support networks amidst demanding caregiving duties.

FM2: I have two daughters, one is five years old, the other is 16, and yes, I also need to spend time with my family. It's not like I only have to attend to my dad and mom.

Finally, the code psychological state probes into emotional and mental well-being, unraveling the intricate layers of subjective experiences linked with the ability of the caregivers to feel able enough to take care of their loved ones. In this case, the same participant reports here experience when she visited her mother at the hospital.

FM2: … [she was] tied to the bed because she would remove the catheter herself, and, um, even that was a bit, yes, traumatic for me because seeing … they didn't warn us, we arrived there and found my mom tied up.

### Positive emotions laden with significance

The second theme surfaces a profound journey with two distinctive codes: hope and gratitude towards Healthcare Providers (HCPs). Hope reflects individuals’ resilient and optimistic outlook amid life-altering experiences, contributing significantly to their transformative quality of life.

FM2: The doctors say that she doesn't meet those necessary parameters, but if I can be honest, I … I have some hope. Well, maybe I'm the daughter, that's why, but I always have hope and … and yes. So, yeah. You also have to live with hope and keep going.

Simultaneously, gratitude towards HCPs continues to be expressed by the FMs, even though the patient has been discharged and is now at home. This underscores deep appreciation for healthcare professionals’ crucial role in this phase, emphasizing the profound impact of their support, care, and expertise. This instills hope even in the post-ICU care.

FM3: I've always believed in you doctors primarily. In fact, everyone tells me that in the intensive care unit the merit is all on the people [the staff], not that this would not be the case in other medical departments, for heaven's sake, but you deal with truly challenging cases, let's say, at the limit. And … and well, you brought her [my sister] back to this world.

This type of narrative, as well as the opportunity to express gratitude towards the doctors, brings out the preciousness of the outcome achieved with the discharge from the intensive care unit, contributing to solidifying the collaboration pact with the doctors and to the transition of roles in the care of the FM.

### Supporting role taken on by a caregiver

The third theme that observed from qualitative interviews centers on the profound ‘Supporting role taken on by a caregiver,’ delineated by four distinct codes. This theme illuminates the multifaceted roles of caregiving, showcasing the diverse ways in which caregivers offer vital support, ranging from practical help to emotional sustenance, ultimately contributing significantly to the overall well-being and quality of life of the individuals they care for. Informational support represents the caregiver's role in providing crucial information and guidance: it involves attending the appointments, taking care of recording medical information, and communicating with the doctors.

FM1: I want to be present, obviously, at all medical appointments, everything – there's also my husband, of course, but primarily me.

Emotional support/Esteem underscores the caregiver's contribution to the emotional well-being of the individual, offering encouragement and fostering a positive self-image. Due to the limited number of occurrences of quotations for esteem (only one was identified), it has been presented alongside the emotional support code. However, it should be recognized that this dimension is particularly important for fostering bonds with the family member, which has protective effects on both. Emotional support often consists of small caring behaviors specifically tailored to the needs and personalities of one's loved ones; an effort that can also strengthen the bond with the family member.

FM23: And when I play music for her … Uh, for example, we interact like this, and so we understand each other a little, you know, between siblings.

Social network support reveals the caregiver's involvement in nurturing and maintaining social connections for the individual. It is an effort aimed at maintaining the social network of the already compromised family member. In doing so, the caregiver has the opportunity to maintain it for themselves as well and find an opportunity for their own support or recreation.

FM2: these wonderful things like video calls exist, thankfully. And then we have video calls every day with the girls, so they see their grandma, eh, they ask her how she's doing, you know.

Tangible support encompasses the practical assistance caregivers provide, addressing the concrete needs of those under their care. It involves fulfilling all the tasks of the person's daily life, from cooking a meal to driving them to the doctor's appointment, but also learning new skills like carefully cleaning and dressing the patient's wounds.

FM5: Mom has a stoma, so basically, I have to change her bag every day because she can't do it for herself […] They nurse taught me there how to deal with the stoma during … They called me one day to give me, like, an accelerated course all in one day, huh. That's where I learned.

The support provided to the FM hinges on these four dimensions, which represent new roles that the caregiver may choose to acknowledge and utilize. The effectiveness of this support and the quality of the ensuing relationship with the family member rely on the caregiver's capacity to identify, undertake, and potentially delegate these responsibilities.

### Life’s transience through the meaning-making of the illness event

The fourth and most existentialist theme delves into ‘Life's transience through the meaning-making of the illness event.’ This profound theme encompasses four intricate codes that offer insights into individuals’ existential reflections. Together, these codes paint a picture of individuals navigating the existential aspects of life in the face of illness. This theme goes beyond the immediate effects of the illness event, delving into the deeper layers of existential contemplation, resilience, and adaptation to an altered life trajectory. Some participants are slightly closer to psychological protection, showing a profound capacity to derive meaning from the transient nature of life, embracing change, and finding purpose even in the midst of challenging circumstances. Cherishing small improvements captures the essence of finding meaning in incremental positive changes during the illness journey, emphasizing the significance of small victories.

FM4: Yeah, and there are days, like today, when we even went for a little stroll at the market.

The code changing my life reflects the transformative impact of the illness event, prompting some participants to re-evaluate and adapt their lifestyles. Participant FM2 shows her change of attitude also towards the disease itself, which in common sense is considered an individual event but in reality, affects the family system.

FM2: Because we always think only about the sick person, but in reality, it's the illness of a person that impacts their whole family.

The code ‘doing everything by myself’ underscores the existential aspect of self-reliance, portraying some family members’ experiences of empowerment and autonomy despite the caregiving challenges, which in some cases leads to a lack of other people to rely on for the care of the patient.

FM5: I don't have support from anyone […] I do everything by myself, I don't have support, I mean there's no one helping me.

Finally, accepting that nothing can be as before investigates into the acknowledgment of irrevocable changes, symbolizing a profound acceptance of the altered reality. Participant FM8 is well aware that the illness event experienced by his husband and herself was highly invasive. She understands that the goal is not to restore previous conditions but to find new dimensions of meaning within the possibilities of the present.

FM8: We try to have a life, yes, I'm not saying like before, because well, you know, things change in such moments, but close enough.

## Discussion

This study sought to examine the post-discharge experiences of family members within a three-month timeframe following the discharge from the ICU. The sample appears to capture a range of familial relationships to the patient, offering a comprehensive view of the individuals involved. The diversity in relationships, spanning from parents and siblings to spouses and children, reflects the complex network of caregivers that may be surrounding the patient.

Measuring QoL as the central aspect of the caregiver experience acknowledges the range of both positive and negative effects that FMs may experience simultaneously. Revisiting the conceptual framework for caregiver quality of life originally proposed by the World Health Organization and later modified by Martin and colleagues (Martin et al., 2021), our findings can also be interpreted through the lens of this model, observing the link between the four variables of physical functioning, social relationships, psychological state, and environment. FMs’ experiences are mapped onto these dimensions to illustrate how different experiences influence overall QoL. The consistency with the framework, as we were able to easily classify the experiences within the dimensions it proposed, and the relationships identified, suggest that this model can be effectively applied in a qualitative study on similar topics. It serves as a guide for analysing findings from lived experiences. In particular, the challenges imposed by the environment (e.g. transportation from hospital to home over long distances or rearranging furniture at home) appeared to influence both the quality and quantity of concrete support that caregivers felt able to provide. A recent study on 72 Taiwanese family caregivers indicated that interventions involving barrier-free home environment improvements could reduce caregivers’ care stress and improve their QoL, in turn indirectly improving interactions and increasing cohesion among family members (Yang et al., 2022).

Moreover, various types of support were identified according to Cutrona and Suhr's model (Cutrona & Suhr, 1992). Such types of social support have been extensively documented in the narratives of diary-style and informative bloggers (Ko et al., 2013). In the real world, roles such as providing tangible assistance, informational support, and network support are most actively embraced by caregivers. From our perspective, the social relationships in the QoL theme are strongly interconnected with caregivers’ perceived ability to provide networking support. In our supposition, given the multitude of diverse needs and, consequently, various forms of support required, a role differentiation would be desirable (Cipolletta et al., 2019). However, what often occurs is that the entire burden falls on one individual, as the assistance provided lacks differentiation. As an example, some studies conducted among dementia patients have indicated that reinforcing social support can mitigate the sensitive risk of burden in informal caregivers of older adults (Nemcikova et al., 2023).

The psychological state of caregiver appeared to be interconnected and reinforced by two particularly impactful emotions ( = hope and gratitude toward HCPs) that we have observed and incorporated into a dedicated thematic area: positive emotions laden with significance. Positive attitudes such as hope and gratitude (and potential trust) can serve as a bridge to foster positive attitudes and enhance overall quality of life (Birkhäuer et al., 2017). Gratitude may exhibit a correlation with the willingness to adhere to instructions, while hope appears to be linked also to a pivotal aspect: cherishing small improvements. These positive emotions towards the staff present pivotal opportunities for intervening and cultivating a robust caregiver-patient-caregiver relationship. This is particularly vital, as the establishment of such a foundation is indispensable for meaningful outcomes (Heyn et al., 2023). Moreover, current perspectives advocate for exploring the potential benefits of intervening on both survivor and caregiver distress following ICU admission, as survivor – caregiver dyads can influence each other’s emotional distress over time (Bannon et al., 2022).

The caregivers’ experience involves two crucial steps. The first is the ability to adjust their expectations regarding what the patient can achieve, and finding the patience to focus on small advances toward an improved QoL for the patient. On the other hand, there is the possibility of accepting that nothing can be as it was before and that, in order to become an effective caregiver, one must also change their own life. A recent research work analysed response from 150 caregivers to an open-ended question concerning their caregiving experiences (Blinka et al., 2022). Participants reported that caregiving provided opportunities for personal growth, learning new skills and a sense of fulfillment and gratitude despite the challenges they encountered. Understanding the changes and transitions associated with caregiving is crucial for enhancing the quality of life of people.

Moreover, changing my life code can manifest in two distinct sides: one facilitating the modification and recalibration of personal expectations (a protective aspect), and the other entailing a restructuring of familial roles, often accompanied by an inclination to assume full responsibility for patient care (a risk factor). A previous study (Cipolletta et al., 2019) pointed out that caregivers who exhibited a greater tendency to rely on the patients and other external resources reported lower levels of depression, anxiety, and strain, in contrast to those who predominantly depended on their own resources. These findings suggest that caregivers benefit more from external support than self-reliance.

There are some key limitations to be acknowledged in relation to the research. Primarily, the study is limited by the partial observation within a brief follow-up period and the single-centre data collection. This means that the study's findings are based on observations made over a short period of time, which may not capture the full extent of changes or developments that occur over a longer timeframe. It is recommended that future research incorporate longitudinal studies to examine changes over time and identify protective and facilitating factors associated with these changes. Among the limitations, it is important to note that telephone interviews were conducted, which inherently reduce the level of interaction compared to face-to-face meetings. However, the interviews were conducted over the phone as many participants resided far from the hospital, and not all participants had the necessary computer skills to support a video call. In addition, the phone call was not scheduled, and perhaps sending the questions to the caregiver beforehand might have allowed for more prepared responses. Then, the phone call might have been affected by a disrupted environment, for example, if there were other individuals around the interviewee or if they were in crowded places. However, this method of data collection was the most effective for an exploratory study with the goal of obtaining information within the specific timeframe of three months post-discharge from ICU. Lastly, the fact that nurses performed the interviews may have influenced the data collected, as participants might have been more or less willing to share certain experiences or perceptions due to their relationship with the interviewers. Additionally, if the interviewers had prior interactions or relationships with the participants, this could have affected the responses, potentially introducing bias or affecting the authenticity of the participants’ narratives.

## Conclusion

In qualitative interviews, participants provided insights into the dynamics and challenges they encountered during the sensitive transition phase of three months after ICU discharge. This study revisits the conceptual framework of caregiver QoL, integrating findings that align with established models and highlighting relationships between variables such as physical functioning, social relationships, mental state, and environment. By emphasizing the presence of positive emotions, particularly towards staff and the care pathway, we identify crucial opportunities for intervention and the development of a strong caregiver-patient relationship. Additionally, exploring the diverse supportive roles that caregivers undertake reveals their specific needs, guiding strategies to support their recovery post-ICU. Ultimately, we recognize that individuals can adapt their personal concepts and reach their full potential, learning to coexist with the demanding caregiving role and achieving a new level of resilience and fulfillment.

## Author contribution

The study was conceptualized by MD, AG, RZ and LE and approved by all authors. Participants were approached by MD, AG, DM, and FV. Interviews were conducted by DM, SG, MR, SG. Data analysis was performed by MD and LE, and the coding scheme was discussed with all the authors. MD and LE drafted and improved the manuscript as all authors critically reviewed the manuscript. Finally, all authors approved the final version of the manuscript.

## Data Availability

Coding of the interviews is available in Italian and can be requested from the corresponding author.
